# The 2023 Impact of Inflammatory Bowel Disease in Canada: Access to and Models of Care

**DOI:** 10.1093/jcag/gwad007

**Published:** 2023-09-05

**Authors:** Holly Mathias, Noelle Rohatinsky, Sanjay K Murthy, Kerri Novak, M Ellen Kuenzig, Geoffrey C Nguyen, Sharyle Fowler, Eric I Benchimol, Stephanie Coward, Gilaad G Kaplan, Joseph W Windsor, Charles N Bernstein, Laura E Targownik, Juan-Nicolás Peña-Sánchez, Kate Lee, Sara Ghandeharian, Nazanin Jannati, Jake Weinstein, Rabia Khan, James H B Im, Priscilla Matthews, Tal Davis, Quinn Goddard, Julia Gorospe, Kate Latos, Michelle Louis, Naji Balche, Peter Dobranowski, Ashley Patel, Linda J Porter, Robert M Porter, Alain Bitton, Jennifer L Jones

**Affiliations:** School of Public Health, University of Alberta, Edmonton, Alberta, Canada; College of Nursing, University of Saskatchewan, Saskatoon, Saskatchewan, Canada; Department of Medicine, University of Ottawa, Ottawa, Ontario, Canada; The Ottawa Hospital IBD Centre, Ottawa, Ontario, Canada; Department of Medicine, University of Calgary, Calgary, Alberta, Canada; SickKids Inflammatory Bowel Disease Centre, Division of Gastroenterology, Hepatology, and Nutrition, The Hospital for Sick Children, Toronto, Ontario, Canada; Child Health Evaluative Sciences, SickKids Research Institute, The Hospital for Sick Children, Toronto, Ontario, Canada; Mount Sinai IBD Centre of Excellence, Division of Gastroenterology and Hepatology, Department of Medicine, Mount Sinai Hospital, University of Toronto, Toronto, Ontario, Canada; Department of Gastroenterology and Hepatology, College of Medicine, University of Saskatchewan, Saskatoon, Saskatchewan, Canada; SickKids Inflammatory Bowel Disease Centre, Division of Gastroenterology, Hepatology, and Nutrition, The Hospital for Sick Children, Toronto, Ontario, Canada; Child Health Evaluative Sciences, SickKids Research Institute, The Hospital for Sick Children, Toronto, Ontario, Canada; ICES, Toronto, Ontario, Canada; Department of Paediatrics, Temerty Faculty of Medicine, University of Toronto, Toronto, Ontario, Canada; Institute of Health Policy, Management, and Evaluation, Dalla Lana School of Public Health, University of Toronto, Toronto, Ontario, Canada; Department of Medicine, University of Calgary, Calgary, Alberta, Canada; Department of Community Health Sciences, University of Calgary, Calgary, Alberta, Canada; Department of Medicine, University of Calgary, Calgary, Alberta, Canada; Department of Community Health Sciences, University of Calgary, Calgary, Alberta, Canada; Department of Medicine, University of Calgary, Calgary, Alberta, Canada; Department of Community Health Sciences, University of Calgary, Calgary, Alberta, Canada; Department of Internal Medicine, Max Rady College of Medicine, Rady Faculty of Health Sciences, University of Manitoba, Winnipeg, Manitoba, Canada; University of Manitoba IBD Clinical and Research Centre, Winnipeg, Manitoba, Canada; Division of Gastroenterology and Hepatology, Mount Sinai Hospital, University of Toronto, Toronto, Ontario, Canada; Department of Community Health and Epidemiology, University of Saskatchewan, Saskatoon, Saskatchewan, Canada; Crohn’s and Colitis Canada, Toronto, Ontario, Canada; Crohn’s and Colitis Canada, Toronto, Ontario, Canada; Department of Community Health and Epidemiology, University of Saskatchewan, Saskatoon, Saskatchewan, Canada; SickKids Inflammatory Bowel Disease Centre, Division of Gastroenterology, Hepatology, and Nutrition, The Hospital for Sick Children, Toronto, Ontario, Canada; Child Health Evaluative Sciences, SickKids Research Institute, The Hospital for Sick Children, Toronto, Ontario, Canada; SickKids Inflammatory Bowel Disease Centre, Division of Gastroenterology, Hepatology, and Nutrition, The Hospital for Sick Children, Toronto, Ontario, Canada; Child Health Evaluative Sciences, SickKids Research Institute, The Hospital for Sick Children, Toronto, Ontario, Canada; ICES, Toronto, Ontario, Canada; SickKids Inflammatory Bowel Disease Centre, Division of Gastroenterology, Hepatology, and Nutrition, The Hospital for Sick Children, Toronto, Ontario, Canada; Child Health Evaluative Sciences, SickKids Research Institute, The Hospital for Sick Children, Toronto, Ontario, Canada; Department of Medicine, McMaster University, Hamilton, Ontario, Canada; SickKids Inflammatory Bowel Disease Centre, Division of Gastroenterology, Hepatology, and Nutrition, The Hospital for Sick Children, Toronto, Ontario, Canada; Child Health Evaluative Sciences, SickKids Research Institute, The Hospital for Sick Children, Toronto, Ontario, Canada; Department of Medicine, University of Calgary, Calgary, Alberta, Canada; Department of Community Health Sciences, University of Calgary, Calgary, Alberta, Canada; Department of Medicine, University of Calgary, Calgary, Alberta, Canada; Department of Community Health Sciences, University of Calgary, Calgary, Alberta, Canada; Crohn’s and Colitis Canada, Toronto, Ontario, Canada; Crohn’s and Colitis Canada, Toronto, Ontario, Canada; Crohn’s and Colitis Canada, Toronto, Ontario, Canada; Crohn’s and Colitis Canada, Toronto, Ontario, Canada; Crohn’s and Colitis Canada, Toronto, Ontario, Canada; Crohn’s and Colitis Canada, Toronto, Ontario, Canada; Crohn’s and Colitis Canada, Toronto, Ontario, Canada; Division of Gastroenterology and Hepatology, McGill University Health Centre IBD Centre, McGill University, Montréal, Quebec, Canada; Departments of Medicine, Clinical Health, and Epidemiology, Dalhousie University, Halifax, Nova Scotia, Canada

**Keywords:** Disease monitoring, eHealth, Integrated care models, Person-centered care, Quality of care

## Abstract

Rising compounding prevalence of inflammatory bowel disease (IBD) (Kaplan GG, Windsor JW. The four epidemiological stages in the global evolution of inflammatory bowel disease. Nat Rev Gastroenterol Hepatol. 2021;18:56–66.) and pandemic-exacerbated health system resource limitations have resulted in significant variability in access to high-quality, evidence-based, person-centered specialty care for Canadians living with IBD. Individuals with IBD have identified long wait times, gaps in biopsychosocial care, treatment and travel expenses, and geographic and provider variation in IBD specialty care and knowledge as some of the key barriers to access. Care delivered within integrated models of care (IMC) has shown promise related to impact on disease-related outcomes and quality of life. However, access to these models is limited within the Canadian healthcare systems and much remains to be learned about the most appropriate IMC team composition and roles. Although eHealth technologies have been leveraged to overcome some access challenges since COVID-19, more research is needed to understand how best to integrate eHealth modalities (i.e., video or telephone visits) into routine IBD care. Many individuals with IBD are satisfied with these eHealth modalities. However, not all disease assessment and monitoring can be achieved through virtual modalities. The need for access to person-centered, objective disease monitoring strategies, inclusive of point of care intestinal ultrasound, is more pressing than ever given pandemic-exacerbated restrictions in access to endoscopy and cross-sectional imaging. Supporting learning healthcare systems for IBD and research relating to the strategic use of innovative and integrative implementation strategies for evidence-based IBD care interventions are greatly needed. Data derived from this research will be essential to appropriately allocating scarce resources aimed at improving person-centred access to cost-effective IBD care.

**Key Points**

1. Quality of IBD care varies across Canada. Crohn’s and Colitis Canada’s Promoting Access and Care through Centres of Excellence (PACE) is a quality improvement initiative that may help standardize quality of care.
2. Patient-identified barriers to accessing care include long wait times, gaps in psychosocial care, treatment and travel expenses, and geographic variation in available specialty care.
3. Many individuals with IBD utilize emergency departments when access to care is limited. However, IBD care in this setting can be inadequate.
4. Several communities, including Indigenous and immigrant communities, and those of lower socio-economic status experience barriers to care.
5. Integrated models of care, including multidisciplinary care teams, may facilitate access to high-quality biopsychosocial care.
6. Multiple timely and appropriate approaches to disease monitoring, including point of care intestinal ultrasound, can support patient-reported outcomes and quality of life. Monitoring strategies should be guided by individual preference and experience.
7. The adoption of virtual healthcare has changed the way IBD care is provided by improving access, provider communication, tracking individual outcomes and timely management of disease and flares, while reducing costs and maintaining individual and clinician satisfaction.

## INTRODUCTION

Approximately 320,000 Canadians currently live with inflammatory bowel disease (IBD), and this number is expected to rise to 470,000 Canadians by 2035 (1). Variation in the availability and structure of IBD care across Canada has been observed (2, 3). This variation has highlighted the need for standardized quality care in IBD (4–7).

Quality in healthcare is defined by the World Health Organization (2022) as “the degree to which health services for individuals and populations increases the likelihood of desired health outcomes (8).” Quality of IBD care is assessed through structures, processes, and outcomes in accordance with the Donabedian Framework (9). Providing high-quality IBD care can reduce geographical variation in care, healthcare service utilization and costs to the health systems, while improving individual health outcomes (6, 9–11). To standardize quality of IBD care, several countries have developed and implemented standardized clinical guidelines and care pathways, including the PACE pathways in Canada (3, 12–16).

**Figure 1.**
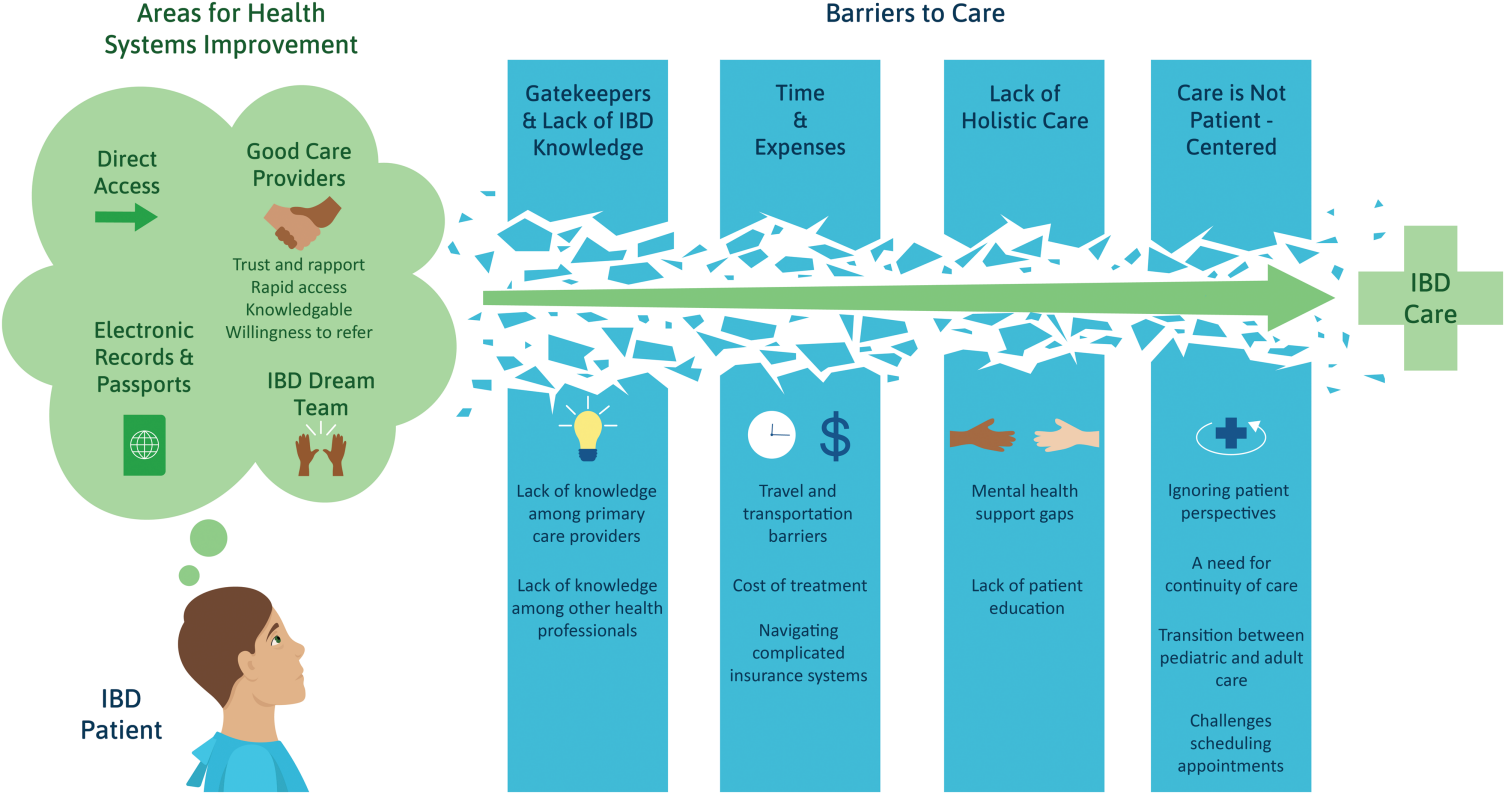
Patient-identified barriers to IBD care in Canada

Access to healthcare has historically been defined as “the fit between the individual and the healthcare system” and is an important aspect of quality IBD care (12). More recently, access has been reconceptualized as a multifaceted experience that moves away from geographic availability of services to include patient-centered considerations such as cultural appropriateness (17). This multifaceted access framework emphasizes the importance of patient-centeredness—a principle that has been at the forefront of IBD health services research in Canada (18–21). Patient-oriented research has established that individuals with IBD and other stakeholders are dissatisfied with existing access to speciality IBD care and allied health professionals (Figure 1). Key barriers to access include wait times, limited access to mental health care that is responsive to the needs of the individual, limited coordination of care, costs, and geographic variation in access to speciality care (19, 22–23). Specific subpopulations, such as pediatric and elderly populations and those residing in rural areas, may face additional barriers to access (4, 5, 21, 24, 25). When access to speciality care is limited, many individuals with IBD access the emergency department (ED); however, the appropriateness of care received in the ED can be inadequate (26, 27). ED utilization is also costly to healthcare systems and efforts have been made to direct people with IBD to other points of care using clinical care pathways (27).

Addressing access to high-quality care is important. Improving quality of care also requires addressing barriers that prohibit the uptake of evidence-based practices and guidelines, including geographic variation in care, limited time and resources, limited data collection, and provider knowledge (11, 28, 29). Some programs have developed and implemented interventions, including novel technology (16, 30–33), IBD nursing roles (16, 34), clinic certifications (30), and open access clinics (35). Further development and adaptation of these patient-oriented, healthcare delivery models in Canadian and locoregional contexts may support improvements in the quality of care provided to persons living with IBD. Improving access to patient-centered, integrated interprofessional care delivery models as well as comprehensive and consistent disease monitoring and treatment strategies are critical; they can be achieved, in part, by leveraging innovations in models of care delivery and eHealth.

## INTEGRATED COLLABORATIVE MODELS OF CARE

Inter-professional and integrated care delivery models that facilitate high-quality care addressing the biopsychosocial needs of persons living with IBD are needed. Integrated models of care (IMC) offer a solution to the fragmented and single system-focus of traditional healthcare delivery (36–38). Globally, IMCs have been implemented for the care of individuals with IBD (39–42).

Integrated care brings together “inputs, delivery, management, and organization of services related to diagnosis, treatment, care, rehabilitation, and health promotion to improve access, quality, user satisfaction, and efficiency (43).” Approaches to the development of IMCs are supported by multiple guidelines, quality standards, and expert opinion (40, 44–46), as well as mounting evidence of the positive impact of IMCs on individuals and health systems (41, 42, 47–49).

An IMC involves a multidisciplinary team (MDT) of healthcare professionals working together to deliver comprehensive care to individuals. Gastroenterologist-led MDTs are more effective than the traditional patient-specialist model of care (42, 49–51). Although robust evidence is lacking in defining IBD MDT membership, guidelines provide some framework including identifying core and ancillary members (12, 15, 44, 52, 53). Core individuals for IBD-specific IMCs include nurses (12–14, 34, 41, 42, 44, 52–75), mental health care providers such as psychologists and psychiatrists (33, 41, 53–55, 57, 62, 63, 66–68, 70, 74, 76–80), dietitians (14, 34, 41, 44, 52, 53, 55, 57, 59, 66–68, 70, 72, 74, 75), and social workers (53, 55, 57, 62, 66, 67, 70). Pathologists, radiologists, and pharmacists with a special interest in IBD have also been suggested as core members of an IMC (44). Ancillary members may also include a rheumatologist, ophthalmologist, hepatologist, dermatologist, pediatric IBD care team, obstetrician, and physiotherapist (33, 44, 52, 53). At a minimum, an IMC team should involve a gastroenterologist lead, a colorectal surgeon, an IBD nurse specialist, a dietitian, and a psychologist or a mental health counsellor. There is little research on best practices for the delivery of IMC, outside of the importance of an MDT; however, one literature review suggests that delivery through standard IBD specialty clinics and virtual care are most common (81).

IMCs are focused on person-centered care, timely access to care, disease education, interprofessional collaboration and communication, and biopsychosocial factors affecting persons with IBD, with the ultimate goal of optimizing IBD care. Improving individuals’ satisfaction and health and population outcomes, while also decreasing costs associated with IBD healthcare is central to IMC program development (42). A prospective cohort study conducted in Australia reported that implementing a formal gastroenterologist-led IBD service reduced hospital admissions, lowering healthcare utilization and costs associated with inpatient care (82). However, costs associated with outpatient service provision were not measured in that study. A Canadian study from Saskatchewan comparing outcomes between individuals exposed and not exposed to an IMC demonstrated that individuals exposed to the IMC had a lower risk of IBD-related surgeries, and for individuals with ulcerative colitis, a lower risk of IBD-related hospitalizations and corticosteroid dependence (41). Perioperative IBD care provided by an MDT was found to provide greater diagnostic accuracy, decreased frequency of elective surgery, decreased disease recurrence, and greater serum albumin and hemoglobin levels (75). A multidisciplinary approach is also recommended for persons with IBD who have additional immune-mediated inflammatory diseases such as spondylarthritis, psoriasis, psoriatic arthritis, or uveitis to allow for a more comprehensive evaluation and treatment approach (71).

The high prevalence of anxiety and depression among persons living with IBD is well documented (83). Untreated mental health disorders have been associated with poor IBD outcomes including more severe IBD symptoms and more frequent flares (84, 85), poor medication adherence (86), higher hospitalization rates (87), and increased healthcare costs (88); therefore, a psychologist or counsellor should be embedded as a core member of the IMC team (42, 44, 50, 51). Additionally, experts and people living with IBD recommend that a psychological assessment be performed for each individual with IBD, rather than just those expressing mental health concerns (39). When needed, IBD specialists should initiate referrals to psychology or have psychotherapists as integrated members of the IBD care team. IMCs that include regular psychological assessments and management of mental health concerns are associated with decreased anxiety, depression, general distress, healthcare resource, opioid and corticosteroid use, as well as improved quality of life (57, 63, 89). However, cost and limited access to psychologists who have expertise in IBD are barriers experienced by individuals with IBD when trying to access this needed care (76). Additional information on mental health and IBD is provided in Graff et al. (in this volume).

There is limited research about the perception of and satisfaction with IMC by persons living with IBD and by healthcare providers (42). Joint provider clinics with a gastroenterologist and surgeon have been seen as satisfactory by individuals because of: decreased anxiety about their IBD, consistent provider communication and decreased numbers of appointments and trips to hospital (61). Many people with IBD do not have access to IMCs, and those who do not have access often feel like they have an unmet healthcare need (10).

Although the benefits of an IMC are now well recognized, several barriers to implementing this care model exist. Mikocka-Walus et al. conducted a multi-national mixed-methods study that included 135 health professionals caring for people with IBD to examine models of care in IBD, including patient-reported barriers to establishing an ideal IBD service (39). The greatest patient-reported barrier was related to short and long-term funding (51%), followed by respondent perception that healthcare systems are not conducive to multidisciplinary care (14%) (39, 42).

## eHealth

The COVID-19 pandemic has brought the importance of virtual healthcare (VC) to the forefront as a viable care delivery strategy to improve access to care. Defined as “an interaction between patients and their healthcare providers that occurs remotely (90),” VC can take place through a variety of communication and technological formats. It can occur synchronously in which both individual and provider are present at the same time with real-time interaction between both parties, typically occurring through video conference or telephone. Conversely, asynchronous VC refers to a patient–provider interaction that occurs and adopts an offline store-and-forward approach. Store-and-forward refers to collecting information that is sent to and assessed by health professionals outside of real-time consultations. Examples include secure emailing or messaging functions that are integrated with electronic medical record systems.

During the COVID-19 pandemic, most persons living with IBD gained exposure to VC (91). A survey by the Canadian Medical Association suggests that individuals were satisfied with VC (92). Nationwide focus groups consisting of people with IBD offered insight into how they experienced VC (93). Telephone-based services were most common; many felt videoconferencing would have improved the interaction. Individuals with IBD identified VC as convenient and feel that it saved them time, especially for short visits. VC visits were especially appealing for those who lived in more remote regions or far from an IBD centre. The main concern about VC expressed by focus group participants was that it may completely replace in-person care. Most participants felt a balance and choice among in-person, video, and phone visits was ideal. Some participants expressed concerns that technology was a potential barrier to accessing VC, especially video-based visits, and emphasized the importance of paying attention to eHealth equity considerations and flexibility of the modality. There is very little guidance in the current literature on how to implement VC for people with IBD that supports equity and patient-centredness, such as how to balance the mode of delivery and potential barriers to access; this is a key knowledge gap that has been identified in research on other health issues (94). A study in the Netherlands developed a comprehensive eHealth implementation guide for healthcare providers and people with chronic myeloid leukemia (95). The guide was co-designed through stakeholder focus groups and interviews to identify key individual, socioeconomic, political, and organizational barriers to eHealth implementation. The co-design of VC implementation guidelines, as well as greater research on key equity issues, could benefit the uptake and accessibility of VC among people with IBD and healthcare providers in Canada.

The adoption of synchronous and asynchronous VC options has had a significant impact on the delivery of IBD care. VC improves access to and communication with physicians, nurses, and other allied healthcare providers (96). Many individuals with IBD and physicians recognize VC modalities as acceptable, feasible, and have expressed their willingness to use these in the future (97). Increases in health care delivery through eHealth platforms has transformed IBD clinical practice patterns. In contrast to the traditional model of in-person care, eHealth can track individuals’ disease activity, medical therapy, and mental health in real time. eHealth can support IBD clinical decision-making through algorithms integrated into electronic medical records allowing for more rapid and timely interventions when managing disease, flares, or mental health concerns (98). Enhanced engagement can also be facilitated by eHealth modalities. Individuals can self-report symptoms or use virtual patient reported outcomes (PROs) systems to track their disease (99). eHealth platforms can facilitate remote monitoring of objective disease measures, such as fecal calprotectin (FC), which may allow for the early detection of IBD flares through point of care assessment via home-based tests that are relayed to and acted upon by the healthcare team (100).

Access to eHealth may also support improved access to specialty care and cost savings for both healthcare systems and individuals with IBD. Several studies have evaluated whether eHealth-facilitated IBD management can improve clinical outcomes. Generally, both individuals with IBD and healthcare providers in Canada have reported that eHealth improves access to specialty care, particularly in underserved communities (101). Reduction in healthcare utilization—in particular, fewer outpatient visits—has consistently been reported in association with eHealth modalities (102–104). However, the impact of eHealth facilitated care delivery on IBD-related hospitalizations and ED visits is less clear (102, 105–107). eHealth tools (e.g., digital applications) may help screen for depression and anxiety leading to prioritization of individuals who need prompt psychosocial support; however, there is varying evidence that VC can reduce psychological distress (104, 108) or improve quality of life (103). Some older studies have found an improvement in adherence to medical therapy, perhaps due to enhanced communication with healthcare providers. However, more recent systematic reviews and a meta-analysis found no benefit of VC on treatment adherence (103, 109). Direct and indirect healthcare cost savings have also been observed in association with eHealth technologies (103, 110, 111). A three-year follow-up Danish register-based study found that although there was a significantly higher cost to enrolling a person with IBD in eHealth compared to standard care (€2,949 vs. €1,621), by year four, eHealth costs became cost neutral or cost-saving to the healthcare system (100). There are also anticipated savings for individuals who may save money by reducing or eliminating travel to in-person appointments (see Kuenzig et al. this volume for more information on indirect and out-of-pocket costs) (100). Although data on the favorable impact of eHealth are promising, there remain inconsistent findings in the literature, highlighting the need for ongoing research relating to eHealth modalities for IBD management.

Limited resources for the set up and maintenance of eHealth platforms and user (individuals with IBD and care providers) accessibility and familiarity remain barriers to equitable access to eHealth. The future of IBD care delivery will likely include a hybrid of both in-person and virtual care. eHealth delivery will continue to evolve and adapt to the needs of individuals and their healthcare providers and is bound to remain an integral part of the IBD model of care moving forward.

## DISEASE MONITORING

The Selecting Therapeutic Targets in IBD (STRIDE-II) guidelines recommend both endoscopic healing and clinical remission, defined by symptomatic relief and improved quality of life (112). Participation and shared decision making are key when defining treatment goals and monitoring the disease course for a condition that is chronically relapsing and remitting (113). For example, although ileocolonoscopy is the gold standard for objective disease monitoring, it is invasive and resource intensive, requires individuals to miss work or school, and requires a purging preparation and sedation, making it the least preferred test for individuals with IBD (114). Access to frequent endoscopic evaluation in many centers in Canada is also limited, particularly in the post-pandemic recovery period (115). An individual’s engagement and participation in decisions around need can improve acceptance of endoscopy (113).

Alternative, non-invasive surrogates for endoscopic activity are routinely used in clinical practice to detect underlying luminal inflammation (116, 117). The most widely used biomarkers include blood-based C reactive protein and stool-based fecal calprotectin (fcal) (118). Although easily repeated for routine monitoring, both tests are non-specific, elevated in concomitant infection or with other sources of inflammation (e.g., diverticulitis), do not reflect disease location nor disease extent, and cannot exclude IBD related complications (118, 119). There is some intra-individual variability in measures of fcal, in addition to falsely elevated measures with commonly used over-the-counter medications such as non-steroidal anti-inflammatory medications (120, 121). Despite these limitations, interval monitoring of biomarkers to objectively detect inflammation beyond symptom control has been shown to improve outcomes and is central to a treat-to-target strategy in IBD (112, 122).

Due to limitations of ileocolonoscopy when assessing small bowel inflammation and disease processes proximal to strictures in Crohn’s disease, additional modalities are often needed to fully stage and grade cross-sectional luminal and extra-luminal disease and complications. Therefore, current guidelines recommend baseline and interval evaluation with cross-sectional imaging, preferably safe, radiation free options such as magnetic resonance enterography (MRE) or intestinal ultrasound (IUS) (123). Although ulcerative colitis has been historically viewed as limited to the mucosa, cross-sectional imaging evidence in acute moderate-to-severe disease states also demonstrates involvement beyond the mucosa, with the submucosa and surrounding mesentery affected (124). Given the presence of significant disease in IBD beyond the endoscopically visible mucosa, the concepts of transmural response and remission have developed as important treatment targets (125). Transmural healing is associated with significant reduction in adverse outcomes including corticosteroid use, hospitalization, and surgery (126, 127).

Access to routine, interval MRE in Canada is challenging due to long wait times resulting in separation between clinical assessment and assessment of disease activity (128). Prior to the COVID-19 pandemic, wait times for medical imaging in most Canadian provinces exceeded the recognized standard of 30 days. As of 2022, it is estimated that most Canadians will wait between 67 and 130 days for medical imaging, depending on the test. Long wait times for medical imaging are linked to limited imaging equipment, few trained technicians, and low government investment (128). MRE requires intravenous and oral contrast. Many individuals prefer to have IUS to monitor their IBD, when given the option (114, 129). IUS has several advantages over MRE, as it can be performed during routine assessment at follow-up in clinic, requires no oral or routine intravenous preparation nor medication (such as motility agents common during MRE), and presents an opportunity for engagement and education (130). Importantly, mounting evidence supports the accuracy of IUS for the detection of both disease activity and complications, when compared to accepted reference standards, including endoscopy and MRE (131–135). In addition, changes in key sonographic parameters can be followed over time, demonstrating responsiveness of IUS to effective medical therapy (124, 136).

There is rising interest in IBD-focused expert provision of IUS to effectively monitor individuals by both providers and individuals with IBD. The International Bowel Ultrasound Group developed an accredited training program, endorsed by the European Crohn’s and Colitis Organization (https://ibus-group.org/). The provision of IUS in Canada is expanding annually, with at least eight expert adult and five pediatric centers currently performing routine IUS for monitoring purposes. IUS provides a person-centered innovation in a treat-to-target monitoring paradigm. Here cross-sectional imaging data (combined with standard bedside assessment, including biomarkers, ileocolonoscopy when indicated, and complementary cross-sectional imaging) contributes to clinical decisions in real time (130, 137–139). This paradigm allows for the timely, appropriate optimization of medical therapy, avoidance of unnecessary corticosteroid use and investigations, in addition to improved individual experience (130, 137–139). The future of IBD monitoring will likely include multiple monitoring approaches including PROs, quality of life, IUS and alternate imaging modalities, and biomarkers, with interval ileocolonoscopy where needed. Monitoring strategies need to be person-centered to ensure reduction of disease burden, damage, and long-term disability.

Although IUS presents a novel innovative, person-centered approach to effectively monitor individuals with IBD, there are several current barriers to widespread adoption and implementation across Canada. The technology required (high end ultrasound machine with specialized transducers) imparts costs beyond costs associated with maintenance and upkeep. These high-quality ultrasound machines are not a usual constituent of IBD-expert clinics in Canada. Moreover, acquisition of IUS expertise and training presents a challenge, with few supports and limited opportunities to acquire adequate training. Finally, system challenges including the lack of provider remuneration for IUS is a barrier to inclusion in busy, high throughput clinics where time constraints limit adoption. This innovation is disruptive, and will require advocacy for change, to optimize individuals’ experience with IBD monitoring.

## CONCLUSION

The rising prevalence of IBD in Canada has underscored the importance of timely and equitable access to speciality care and allied health professionals through IMCs. Improved access to care is associated with better health outcomes, yet there remains variation in access and quality of care across Canada. Implementing and evaluating standardized quality care indicators in clinical practice, such as the PACE clinical care pathways, may support access to care and improve health outcomes. Key focus areas include the implementation of IMCs and leveraging innovative eHealth platforms to support enhanced communication, implementation of evidence-based care, and multiple disease monitoring approaches that center individuals’ quality of life and supports individual decision-making and outcomes.

Limitations in uptake and implementation of evidence-based clinical care guidelines and structural challenges, including system design and limited funding, highlight just a few of the behavioral and environmental barriers to high-quality care provision. Moving forward, attention must focus on leveraging innovative eHealth technology and models of care to improve equitable, person-centered access to and delivery of evidence-based care. Potential interventions to address specific barriers to access experienced by subpopulations, such as pediatric and those in rural locales, must also be further explored.

## KNOWLEDGE GAPS AND FUTURE RESEARCH DIRECTIONS

1. There is limited knowledge on the diverse lived experiences of IBD and access to services from many sub-populations, including those living in rural, remote, and Northern communities; transgender and gender diverse individuals; Indigenous peoples; and other equity-deserving groups.
2. Many individuals continue to utilize emergency departments as a point of access to IBD care; however, there is limited knowledge on whether some sub-populations have greater reliance on emergency departments and how to support their care needs.
3. Person-oriented healthcare delivery interventions should also be evaluated for effectiveness in facilitating access to IBD care for sub-populations.
4. Next steps should involve addressing barriers to consistent physician uptake of clinical guidelines and further developing and adapting person-oriented healthcare delivery interventions (virtual and in-person) specific to Canada to support access.
5. Virtual healthcare should evolve and adapt to the needs of the provider and to the needs of the individuals it is servicing.

## PATIENT AND CAREGIVER PARTNER PERSPECTIVE

Access to healthcare for individuals with IBD is reconceptualized as a multi-faceted experience that incorporates person-centered considerations. Patient partners recognize that barriers to accessing care remain, including lengthy wait times, limited access to mental health care, limited coordination of care, out of pocket costs, and geographic challenges. Improved access to quality medical care, including early diagnosis and treatment options for those living with IBD are encouraged. Patient partners frequently experience communication gaps between health care providers, such as challenges with connecting to dietitians and mental health concerns, and they recognize that IBD related care is often siloed. Integrated, multidisciplinary, collaborative models of care can improve communication with and between healthcare providers, enhance access to care, and address the biopsychosocial needs of individuals living with IBD. Virtual care delivery is welcomed by patient partners as a strategy to enhance access to care and can result in a reduction in healthcare utilization, direct and indirect health care cost savings, and improved quality of life for persons living with IBD. An individualized, hybrid (in person and virtual) care delivery approach is deemed as ideal by patient partners. eHealth platforms can increase individuals’ engagement in their care, enhance remote monitoring, and improve communication between individuals living with IBD and healthcare providers.

## POLICY IMPLICATIONS AND KEY ADVOCACY OUTCOMES

1. Increased government funds should be invested in interventions that improve access to care (e.g., increased staffing in under-resourced areas, augmented psychosocial supports, eHealth supports, patient navigators, peer supports).
2. Universal healthcare coverage should be expanded to include out-of-pocket treatment expenses that limit access to care.
3. Quality improvement initiatives (e.g., PACE) should be embedded in institutional policies and practices to support standardized quality care regardless of geographic locale.
4. Crohn’s and Colitis Canada should advocate for improved access to care for all persons with IBD, but particularly for equity-deserving populations who face the greatest number of barriers to care, such as Indigenous peoples. Different care pathways should be developed to address the specific care needs of different sub-populations.
5. Crohn’s and Colitis Canada should advocate for increased education of IBD in medical school curricula and as part of ongoing professional development for physicians and healthcare professionals.
6. Advocacy for access to care should incorporate policies that support quality of life for people living with IBD (e.g., accommodations/supports in the workplace and school, public restroom access).
7. Governments must continue to support virtual care, including tele-medicine and video appointments, to enable care to remote populations or those who cannot physically travel to specialist clinics.

## SUPPLEMENT SPONSORSHIP

This article appears as part of the supplement “The Impact of Inflammatory Bowel Disease in Canada in 2023”, sponsored by Crohn’s and Colitis Canada, and supported by Canadian Institutes of Health Research Project Scheme Operating Grant (Reference number PJT-162393).

## Data Availability

No new data were generated or analyzed in support of this review.
